# Is Hybridization a Source of Adaptive Venom Variation in Rattlesnakes? A Test, Using a *Crotalus scutulatus* × *viridis* Hybrid Zone in Southwestern New Mexico

**DOI:** 10.3390/toxins8060188

**Published:** 2016-06-16

**Authors:** Giulia Zancolli, Timothy G. Baker, Axel Barlow, Rebecca K. Bradley, Juan J. Calvete, Kimberley C. Carter, Kaylah de Jager, John Benjamin Owens, Jenny Forrester Price, Libia Sanz, Amy Scholes-Higham, Liam Shier, Liam Wood, Catharine E. Wüster, Wolfgang Wüster

**Affiliations:** 1 Molecular Ecology and Fisheries Genetics Lab, School of Biological Sciences, Bangor University, Bangor LL57 2UW, UK; giulia.zancolli@gmail.com (G.Z.); bsu057@bangor.ac.uk (T.G.B.); axel.barlow.ab@gmail.com (A.B.); bsu069@bangor.ac.uk (R.K.B.); kimc@live.co.uk (K.C.C.); bsue65@bangor.ac.uk (K.D.J.); bsu052@bangor.ac.uk (J.B.O.); jennyp@live.co.uk (J.F.P.); amy.scholes.higham@gmail.com (A.S.-H.); bsu049@bangor.ac.uk (L.S.); woodlc08@hotmail.com (L.W.); c.wuster@me.com (C.E.W.); 2 Evolutionary and Adaptive Genomics Group, Institute for Biochemistry and Biology, University of Potsdam, Karl-Liebknecht-Str. 24-25, Haus 29, 14476 Potsdam (Golm), Germany; 3 Venomics and Structural Proteomics Laboratory, Instituto de Biomedicina de Valencia, Consejo Superior de Investigaciones Científicas (CSIC), Jaume Roig 11, 46010 Valencia, Spain; jcalvete@ibv.csic.es (J.J.C.); libia.sanz@ibv.csic.es (L.S.); 4 Chiricahua Desert Museum, P.O. Box 376, Rodeo, NM 88056, USA

**Keywords:** adaptation, *Crotalus*, evolution, hybridization, introgression, Mojave toxin, molecular evolution, venom

## Abstract

Venomous snakes often display extensive variation in venom composition both between and within species. However, the mechanisms underlying the distribution of different toxins and venom types among populations and taxa remain insufficiently known. Rattlesnakes (*Crotalus*, *Sistrurus*) display extreme inter- and intraspecific variation in venom composition, centered particularly on the presence or absence of presynaptically neurotoxic phospholipases A_2_ such as Mojave toxin (MTX). Interspecific hybridization has been invoked as a mechanism to explain the distribution of these toxins across rattlesnakes, with the implicit assumption that they are adaptively advantageous. Here, we test the potential of adaptive hybridization as a mechanism for venom evolution by assessing the distribution of genes encoding the acidic and basic subunits of Mojave toxin across a hybrid zone between MTX-positive *Crotalus scutulatus* and MTX-negative *C. viridis* in southwestern New Mexico, USA. Analyses of morphology, mitochondrial and single copy-nuclear genes document extensive admixture within a narrow hybrid zone. The genes encoding the two MTX subunits are strictly linked, and found in most hybrids and backcrossed individuals, but not in *C. viridis* away from the hybrid zone. Presence of the genes is invariably associated with presence of the corresponding toxin in the venom. We conclude that introgression of highly lethal neurotoxins through hybridization is not necessarily favored by natural selection in rattlesnakes, and that even extensive hybridization may not lead to introgression of these genes into another species.

## 1. Introduction

Variation in venom composition is a ubiquitous phenomenon in venomous snakes at all taxonomic levels, from temporal variation within individuals to higher levels. [1,2]. The often extensive compositional variation between conspecific populations or between closely related species has been of particular interest, partly due to its medical consequences [3,4,5] and partly due to its potential as a model system for understanding adaptive evolution at the molecular level (e.g., [6,7,8]): many species display extreme intraspecific geographic variation in venom composition, and this variation may bear little relationship to population genetic structure or organismal phylogeny [9,10]. Natural selection for optimization of venom to the diet of snakes has been identified as a likely key driver of venom evolution in several groups [9,11,12,13]. However, these examples consist primarily of snakes with extreme dietary variation and/or disjunct distributions (e.g., *Calloselasma rhodostoma*), or groups of well-differentiated species (e.g., *Micrurus*, *Echis*), and the forces underlying venom variation in other cases remain poorly understood [10,14,15]. Moreover, the genetic mechanisms underlying variation in snake venom composition, and the distribution of individual toxins among populations and species, remain largely unknown.

Rattlesnakes (genera *Crotalus* and *Sistrurus*) constitute an excellent group of model organisms for the study of venom variation, as they display extensive inter- and intraspecific variation in venom composition [16]. An underlying theme in *Crotalus* appears to be the presence of alternative and often largely mutually exclusive envenoming strategies: type I venoms [16] contain large amounts of snake venom metalloproteinases (SVMPs), whereas type II venoms contain a high concentration of presynaptically neurotoxic, heterodimeric PLA_2_ toxins such as crotoxin and Mojave toxin (MTX) [17,18], and are typically considerably more lethal in the mouse model than their type I counterparts.

Remarkably, the distribution of these different strategies among rattlesnakes shows little congruence with phylogeny or even species limits. Both strategies can be found across the full phylogenetic breadth of rattlesnakes. Species showing intraspecific variation, with different conspecific snakes secreting either type I and type II venoms [5,14,19,20,21,22,23,24], can be found in all major rattlesnake clades [16]. This intraspecific variation can be ontogenetic, such as in *C. simus*, where the venom changes from type II to type I during ontogeny [25,26], or geographic, in species such as *C. scutulatus* and *C. horridus*, where both venom types occur in different parts of their distributional range [14,21,27]. In at least some cases, such as *C. scutulatus*, this variation appears to be related to the presence or absence of the genes encoding these toxins rather than gene expression [22,28,29,30].

Mapping these apparently homologous toxins onto the phylogeny of rattlesnakes would require remarkable numbers of gene loss events, or, even less plausibly, astonishingly numerous instances of convergent evolution. An alternative explanation for these patterns, which bypasses this difficulty, is adaptive hybridization. Hybridization has long been flagged as a potential source of adaptive variation and innovations [31,32]. Extensive studies of hybrid zones between closely related species or differentiated conspecific populations have demonstrated that selectively advantageous genes are able to cross hybrid zones and spread into the other species, provided they are not linked to deleterious alleles at other loci [33,34,35]. This applies even with slight reductions in hybrid fitness, which cause these zones to act as sinks for selectively neutral alleles [33,36].

The hypothesis of a hybridogenic origin of the startling parallel patterns of intraspecific venom variation in several rattlesnakes holds considerable intuitive appeal: by effectively providing a shortcut for gene transfer between the branches of rattlesnake phylogeny, introgressive hybridization would explain geographic variation in the presence of particular gene orthologs in multiple unrelated species more parsimoniously than any hypothesis based solely on phylogenetic relationships. Glenn and Straight [37] suggested that the presence of MTX-like toxins in some individuals of *Crotalus viridis* (Prairie rattlesnake) from southwestern New Mexico was due to hybridization with nearby populations of type II venom *C. scutulatus*. Aird *et al.* [38] noted the resemblance between the venoms of *C. atrox* and type I venom *C. scutulatus*, and suggested that the type I venoms of some *C. scutulatus* populations may be due to past hybridization between the two species. More recently, the presence of neurotoxic PLA_2_ toxins in some populations of *C. horridus* has been variously attributed to past hybridization with *Crotalus scutulatus* [39] and *Sisturus catenatus* [30]. Similar arguments have been made to explain the presence of neurotoxic PLA_2_ toxins in some European vipers [40,41]. However, none of these studies provided any independent evidence of interspecific hybridization having taken place. Moreover, despite this interest in hybridization as a possible mechanism for venom variation, we are not aware of any published study rigorously examining venom composition across a well characterized hybrid zone in any venomous animal.

Interspecific hybridization, potentially as a result of anthropogenic disturbance, has also been invoked in the popular literature to explain a claimed increase in the clinical severity of rattlesnake bites in the USA [42]. The evidence for this hypothesized increase was robustly deconstructed by Hayes and Mackessy [43]. However, the intriguing question remains whether occasional hybridization events could result in the rapid spread of novel, selectively advantageous toxin genes through the gene pool of a different species. Given their radical effect on venom function and lethality, one could hypothesize that highly lethal toxins such as MTX might be especially prone to this form of introgression and subsequent selective sweeps.

It is an implicit assumption of any hypothesis of hybridogenic introgression of venom toxin genes that the introgressing genes confer a selective advantage to the receiving gene pool [33,35]. By the same token, any true hybrid zone between rattlesnake species with different venom compositions would thus provide a test of the hypothesis that particular toxins could be highly selectively advantageous and could spread rapidly across species limits after hybridization. Since individual variation in venom composition can lead to differential venom effectiveness against different prey species [44], and thus to potential differences in individual fitness, this scenario seems potentially feasible.

Although numerous individual hybrids between different species and even genera of rattlesnakes have been documented [45,46,47,48,49,50], the frequency and importance of hybridization have been disputed [51]. The present study was prompted by the discovery of multiple specimens phenotypically intermediate between the Mohave rattlesnake (*Crotalus scutulatus*) and the Prairie rattlesnake (*C. viridis*) in a contact zone along the eastern slope of the Peloncillo Mountains, Hidalgo County, New Mexico, where the two species are largely parapatric [52]. This region corresponds approximately to the location from which Glenn and Straight [37] reported MTX-secreting specimens of *Crotalus viridis*, which they interpreted as evidence of hybridization with neighboring populations of type II venom *C. scutulatus*.

A third large rattlesnake species, the western diamondback (*C. atrox*), occurs sympatrically with both species in Arizona and New Mexico, and also across the hybrid zone. Hybridization between *C. scutulatus* and *C. atrox* has been suspected of shaping venom composition in the former [37]. Moreover, a few individuals in the putative *C. scutulatus* × *viridis* hybrid zone were visually intermediate between *C. scutulatus* and *C. atrox* rather than *C. scutulatus* and *C. viridis*. We therefore included all three large, sympatric rattlesnake species from the area in this assessment of hybridization and its effects on venom composition.

Here, we use this apparent hybrid zone to test the hypothesis that highly lethal neurotoxic PLA_2_ toxins are likely to introgress into the gene pool of species lacking them. We analyze morphological data, mitochondrial DNA and single-copy nuclear gene sequences to test for evidence of hybridization between *C. scutulatus* and *C. viridis* or *C. atrox*. We then test for the presence of the genes encoding the acidic and basic subunits of the Mojave toxin using a PCR-based assay and sequencing, and relate the presence or absence of the toxin to the hybrid status of the snakes. Finally, we verified the presence of MTX in the venom by reverse-phase high performance liquid chromatography (RP-HPLC) to establish a link between hybrid status, toxin genotype and venom phenotype.

## 2. Results

### 2.1. Morphology

**Figure 1:**
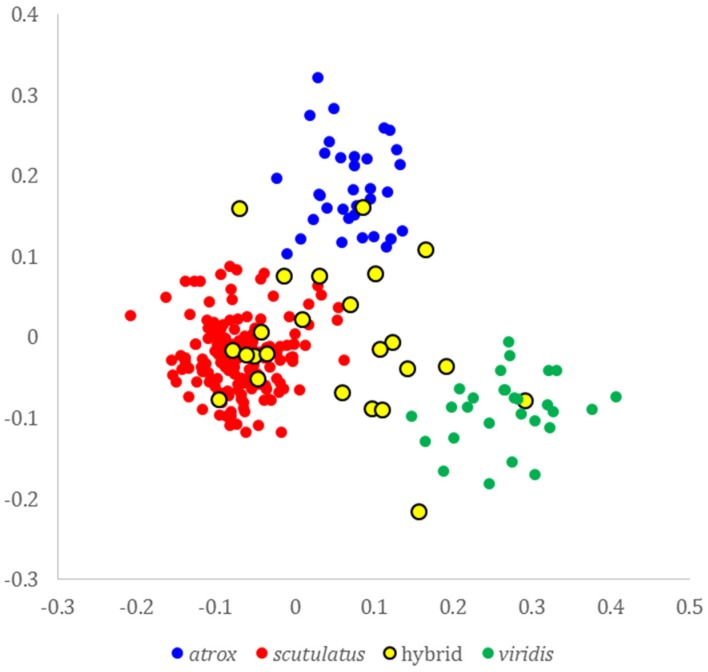
Ordination of specimens of *C. scutulatus*, *C. atrox*, *C. viridis* and putative hybrids along the first two axes of a principal components analysis of nine morphological characters. All specimens from the eastern slope of the Peloncillo Mountains in southwestern New Mexico are labeled as hybrids irrespective of morphological or genetic profile. The first and second principal components represent 41.5% and 23.7% of the total variance, respectively.

Principal coordinates analysis of nine characters of head scalation and tail coloration revealed clearly distinct clusters representing the three species *C. atrox*, *C. scutulatus* and *C. viridis*, with a number of phenotypically intermediate specimens originating from the *C. scutulatus*—*C. viridis* contact zone in Hidalgo County, New Mexico. The analysis revealed both specimens intermediate between *C. scutulatus* and *C. atrox*, and between *C. scutulatus* and *C. viridis*, but not between *C. atrox* and *C. viridis* (Figure 1).

### 2.2. Molecular Evidence of Hybridization

**Figure 2:**
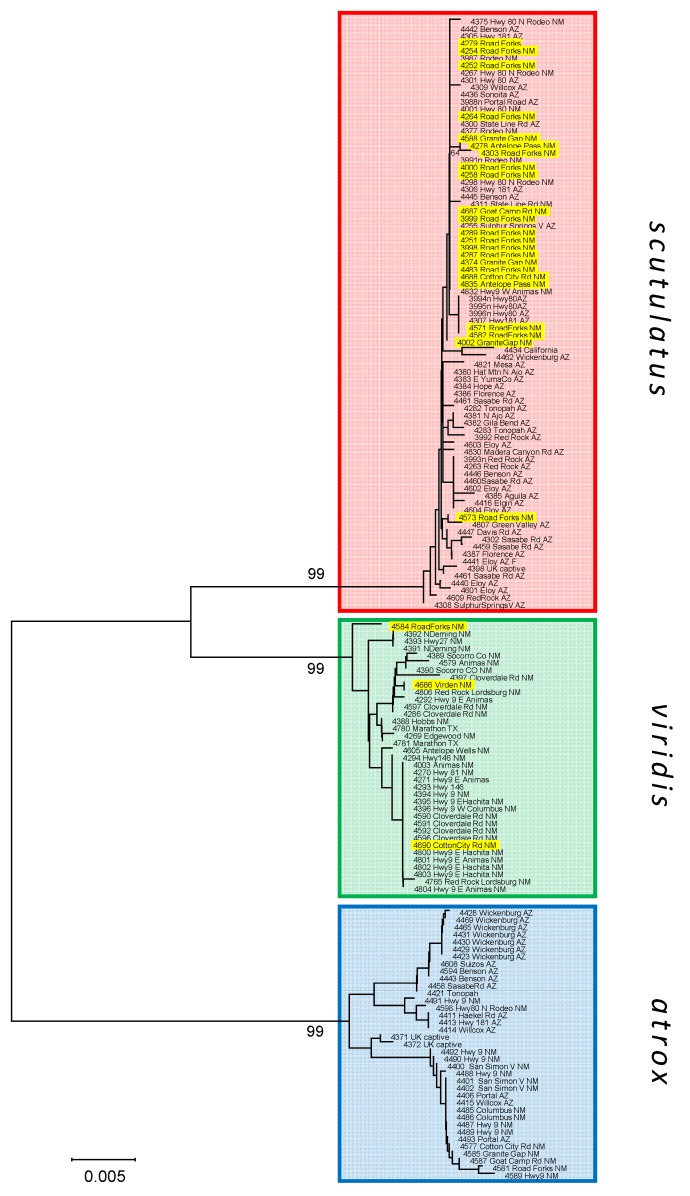
Neighbor-joining tree of individual ND4 sequences. Tip labels with yellow background indicate specimens from the putative hybrid zone in SW New Mexico.

We obtained sequences of the mitochondrial NADH subunit 4 (ND4) gene and four single-copy nuclear genes (NT3, R35, SELT, ETS) from 156 specimens (40 *C. atrox*, 56 *C. scutulatus*, 34 *C. viridis* and 26 specimens from the putative *C. scutulatus* × *viridis* hybrid zone). Information on our sequence alignments is shown in Table 1. A neighbor-joining tree obtained from the mitochondrial ND4 sequences (Figure 2) grouped all specimens into three clusters corresponding to the three species under study, with 99% bootstrap support for the monophyly of the haplotypes of each species. The vast majority of specimens from the putative hybrid zone carried *C. scutulatus* haplotypes, and a few *C. viridis* haplotypes. No putative hybrid haplotypes clustered as *C. atrox*. Mitochondrial sequences and phased haplotype sequences of all nuclear genes are available under GenBank accession numbers KX256288-KX257025.

**Figure 3:**
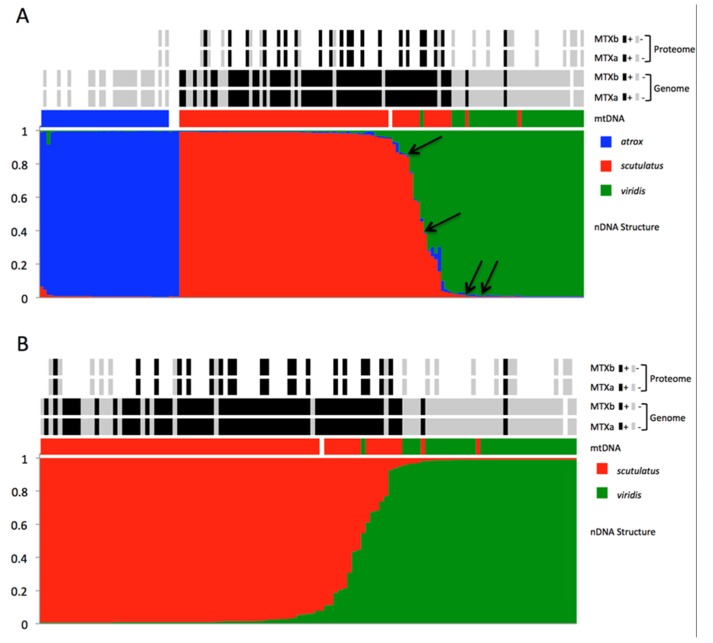
(**A**) Bayesian population clustering of individuals of *C. atrox*, *C. scutulatus*, *C. viridis* and putative hybrids based on allele frequencies of four single copy nuclear genes. Above the Structure clustering, rows of boxes indicate mtDNA haplotype affinities (same colors as nuclear structuring), and the presence (black) or absence (grey) of the genes coding for the basic and acidic subunits of Mojave toxin (MTX), and above the confirmed presence or absence of the corresponding proteins in the venom. White spaces indicate absence of data. Black arrows indicate specimens morphologically intermediate between *C. scutulatus* and *C. atrox*. (**B**) Equivalent analysis excluding *C. atrox* to emphasize hybrid zone between *C. scutulatus* and *C. viridis*.

Bayesian clustering analysis of all three species using Structure 2.3.4 and *K* = 3 showed extensive evidence of admixture between *C. scutulatus* and *C. viridis*, but virtually no evidence of hybridization between *C. atrox* and *C. scutulatus* or *C. viridis* (Figure 3A). Only one predominantly *C. viridis* specimen from the hybrid zone showed some evidence of admixture from both *C. atrox* and *C. scutulatus*. Specimens morphologically intermediate between *C. scutulatus* and *C. atrox* were identified genetically as being *C. scutulatus* × *viridis* or pure *C. viridis*, but in any case without admixture from *C. atrox* (Figure 3A).

Structure analysis of *C. scutulatus* and *C. viridis* individuals only, and K = 2, showed a continuum of levels of admixture (Figure 3B). Virtually all specimens from outside the immediate vicinity of the hybrid zone showed less than 5% admixture from the other species, although the demarcation was sharper in *C. viridis* than *C. scutulatus*. Among admixed specimens, 14 displayed species membership (Qi) values between 0.7 and 0.95, and seven Qi values between 0.5 and 0.7, indicating the presence of backcrosses as well as F1 hybrids. Most of the admixed individuals had *C. scutulatus* mtDNA haplotypes, as did two specimens of *C. viridis* without evidence of admixture from near the main hybrid zone.

In all Structure analyses, independent and correlated frequency models generated functionally identical results. Only the former are presented here.

### 2.3. Detection of Mojave Toxin

**Figure 4:**
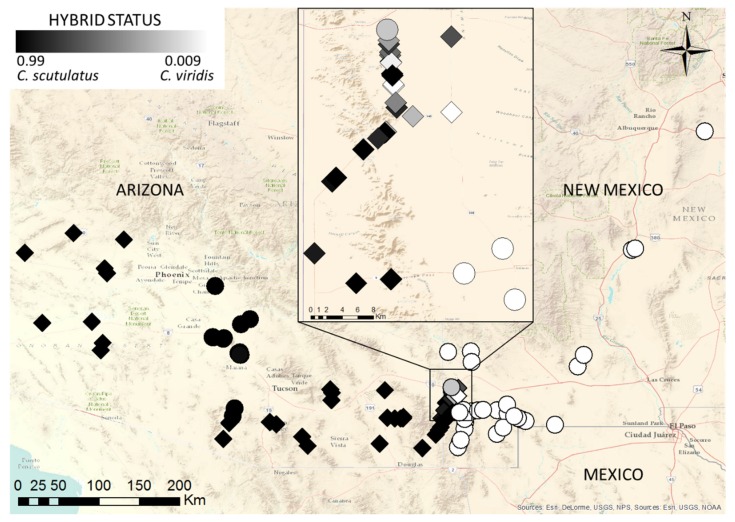
Sampling localities, hybrid status and MTX status of individuals of *C. scutulatus*, *C. viridis* and their hybrids. Diamonds indicate MTX+ve, circles MTX−ve individuals, the degree of shading of the symbols indicates the proportion of the genome attributed to *C. scutulatus* in the Structure analysis.

Newly designed primers for the acidic and basic subunits of MTX (MTXa and MTXb, respectively) produced fragments of 1246 b.p. of MTXa and 1266 b.p. of MTXb, including exon 3 and introns 2 and 3, as well as small stretches of exons 2 and 4. The two MTX subunits were strictly linked: all specimens provided evidence of the genes for either both or neither of the subunits. We did not find any individuals with only one subunit. All partial sequences of the acidic and basic subunits were identical to or differed by no more than three (MTXa) and seven (MTXb) base pairs from the published sequence [53] (GenBank accession numbers U01026-7). Individuals of *C. scutulatus* lacking the MTX subunit genes were all from the documented zone of type I venom snakes in Central Arizona [21,54] (Figure 4). Within the hybrid zone, all MTX-positive individuals were either genetically pure *C. scutulatus* or showed evidence of admixture, either through their scnDNA profiles or possession of *C. scutulatus* mitochondrial haplotypes (Figure 3). The sole exception was an MTX-positive specimen of *C. viridis* without evidence of admixture from approximately 5 km east of a large number of MTX-positive admixed specimens. We did not find any MTX-positive *C. viridis* from locations more distant from the contact zone (Figure 4). None of the included *C. atrox* tested positive for either MTX subunit.

**Figure 5:**
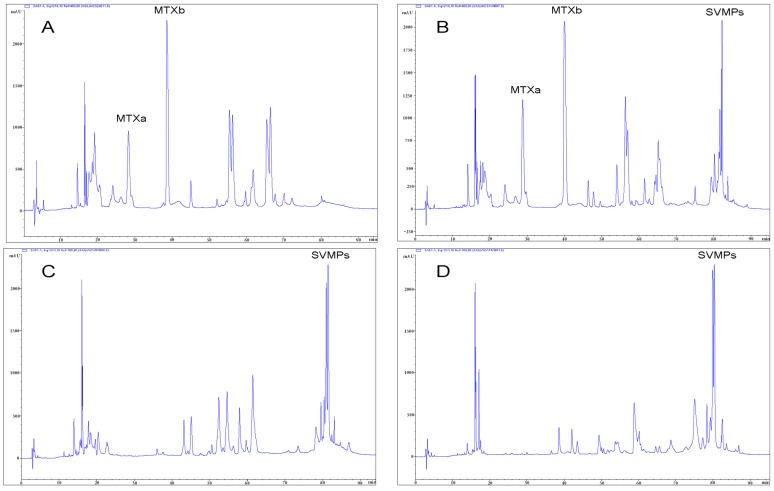
Examples of reverse-phase high performance liquid chromatography (RP-HPLC) chromatograms from different venoms included in the study. A. *Crotalus scutulatus* type II venom with MTX but lacking snake venom metalloproteinases (SVMPs); sample 4311, nr. Rodeo, Hidalgo Co., NM. B. *Crotalus scutulatus* × *viridis*, hybrid containing MTX and SVMPs; sample 4687, nr. Cotton City, Hidalgo Co., NM. C. *Crotalus viridis*, typical venom lacking MTX but containing SVMPs; sample 4590, nr. Animas, Hidalgo Co., NM. D. *Crotalus atrox*, typical venom lacking MTX but containing SVMPs; sample 4594, nr. Benson, Cochise Co., AZ.

We obtained RP-HPLC profiles of the venoms of 41 snakes (two *C. atrox*, 20 *C. scutulatus*, 11 *C. viridis*, and eight putative hybrids). The characteristic peaks of the two MTX subunits [5] were unequivocally recognizable (Figure 5). Consistent with the genomic data, all examined venoms contained either both MTX subunits or neither. No venom showed evidence of only a single peak. Combining proteomic and genomic results revealed a strict linkage between genotype and phenotype: all available venoms from snakes with the MTX subunit genes displayed the two characteristic peaks corresponding to the basic and acidic subunits of MTX, whereas no individual lacking the genes produced them in the venom (Figure 3).

## 3. Discussion

In this study, we set out to test the hypothesis that interspecific hybridization would facilitate introgression by highly lethal presynaptic neurotoxin genes such as Mojave toxin, and thus lead to evolutionary changes in the venom composition of the receiving species. Our results do not support this hypothesis in this instance.

Our genetic analyses unequivocally demonstrate the existence of a narrow hybrid zone between *Crotalus scutulatus* and *C*. *viridis* along the eastern slope of the Peloncillo Mountains in Hidalgo County, southwestern New Mexico. Many, but not all, the specimens in the main hybrid area (Highway 80, within 18 km South of Road Forks, Hidalgo Co.) are recovered as genetically admixed in Structure analyses. Equally, many are morphologically intermediate between *C*. *scutulatus* and *C*. *viridis*. Our single copy nuclear gene data show a continuum of levels of admixture between *C*. *scutulatus* and *C. viridis*, indicating that many of the specimens are the result of backcrosses with either parental species. F1 hybrids between the two species are thus fertile and able to breed with either parent stock. Despite some morphological indications to the contrary, we found little genetic evidence of hybridization between *C. atrox* and either *C. scutulatus* or *C. viridis*. Individual specimens that were morphologically intermediate between *C. scutulatus* and *C. atrox* showed no genetic evidence of admixture from *C. atrox*, but appear to be *C. scutulatus* × *viridis* hybrids or backcrosses. Their phenetic resemblance to *C. atrox* is due to a tail pattern involving bands of greater width than found in *C. viridis* and equal width of dark and light bands, as found in *C. atrox*. So far, this is only the second genetically characterized hybrid zone between two venomous snake species (after the *Vipera aspis* × *latastei* hybrid zone in northern Spain, [55]).

Our results shed new light on the genetics of the MTX subunits. Contrary to Wooldridge *et al.* [28], we invariably found the genes encoding the basic and acidic subunits of MTX to be either both detectable or both absent in all specimens. Wooldridge *et al.* [28] reported that type I venom snakes lack only the gene encoding the acidic subunit, but retain the gene for the basic subunit. We were unable to replicate that result in our much larger sample. We suspect that their detection of the basic subunit gene in type I venom specimens may have been due to non-specific cross-amplification of other PLA_2_ toxins by their primer set: we obtained sequences of PLA_2_-like genes differing from the MTX basic subunit when using Wooldridge *et al.*’s basic subunit primers in specimens from the type I venom zone. Our redesigned primers (see methods) did not incur this problem, and all sequence-confirmed positive PCRs were identical to within a few base pairs with the published MTX sequences [53]. These data suggest that the two subunits are tightly linked in the genome of *C. scutulatus*, presumably due to close proximity on the same chromosome.

Furthermore, our results demonstrate an absolute genotype-phenotype link for the two MTX subunit genes: every single tested specimen with positive PCR results for the MTX subunits yielded HPLC profiles with the two characteristic peaks corresponding to the MTX subunits [5], whereas no specimen without the genes yielded those peaks. This applied independently of genetic profile or hybrid status, and demonstrates that genotypic differences are reflected in the phenotype, and thus provide potential targets for natural selection. This situation contrasts with that found in other viperids, e.g., *Echis*, in which selective transcription and translation as well as post-translational modifications play a prominent part in shaping venom composition [56].

Both subunits of MTX were present in most *C. scutulatus* (except from the previously documented area of type I venom snakes in central Arizona, [21]), but absent from most *C. viridis*. In southwestern New Mexico, only genetically “pure” *C. scutulatus* or individuals with clearly admixed genotypes were positive for both MTX subunits, with the exception of three specimens identified as genetically pure *C. viridis* based on scnDNA markers that were positive for the MTX subunits. Two showed evidence of admixture in the shape of *C. scutulatus* ND4 haplotypes, and all three were from the immediate vicinity of the hybrid zone and surrounded by other admixed specimens. Overall, there is no evidence of introgression of MTX genes into the genome of *C. viridis* beyond the hybrid zone and its immediate vicinity. We suspect that the snakes identified as Mojave toxin-bearing *C. viridis* by Glenn and Straight [37] were in reality hybrids from the zone documented here, although this cannot be verified in the absence of precise locality information in that paper.

The absence of either subunit of MTX in any of our *C. atrox* is consistent with the lack of admixture between this species and the others, as well as most of the literature on the species, although Minton and Weinstein [57] did report low concentrations of MTX from a few specimens of *C. atrox*. The lack of admixture between *C. atrox* and *C. scutulatus* in our data also argues against a role for hybridization between these two species in generating the type I venom population of *C. scutulatus* in Central Arizona, as hypothesized by Aird *et al.* [38]. None of our MTX-negative *C. scutulatus* showed evidence of admixture from *C. atrox*, and neither did any other *C. scutulatus* in our sample, a result consistent with previous analyses [51].

The failure of the MTX genes to spread into the range of *C. viridis* argues against the hypothesis that highly lethal neurotoxins necessarily represent a strong adaptive advantage for rattlesnakes. The parapatry between these two closely related species of rattlesnakes represents a best-case scenario for adaptive introgression of toxin-encoding genes: other things being equal, closely related species are more likely to be reproductively compatible than distantly related species, hybrids are likely to incur a lower loss of fitness than hybrids between more distantly related species, and it is less likely that linked, selectively disadvantageous loci will slow the spread of advantageous toxin genes [34,35]. The imperviousness of the gene pool of *C. viridis* to penetration by the MTX genes thus suggests that possession of these highly lethal neurotoxins is not necessarily a strong selective advantage. Clearly, we cannot exclude the possibility of a different outcome under other circumstances, such as different selective regimes or different constellations of linked genes. Moreover, since we do not know the age of this contact zone, we cannot reject the possibility that, given sufficient time, a degree of introgression of MTX genes may occur. However, given the results presented here, we conclude that the *C. scutulatus* × *viridis* hybrid zone does not provide evidence favoring the hypothesis that limited hybridization may be enough to facilitate the wider and relatively rapid spread of toxin genes in a species in which they were previously missing.

Hybridization has been invoked as a cause of venom variation in multiple groups of snakes [30,38,39], but with little evidence beyond incongruence between phylogeny and venom composition. The rigorous genetic identification of additional hybrid zones between venomous snake species could make a significant contribution to our understanding of venom evolution and the role of adaptive hybridization therein. Until then, based on the results presented herein, we suggest that hybridization should not be invoked as an explanation for unexpected patterns of inter- and intraspecific variation in snake venom composition or for unusual cases of clinical snakebite envenoming without compelling evidence.

## 4. Materials and Methods

### 4.1. Morphological Methods

From digital photos of 245 field-captured specimens, we recorded nine characters of head scalation and tail pattern (Table 2). We used the software MVSP 3.2 (Kovach Computing Services, Pentraeth, UK, 2007) to perform a Principal Components Analysis after data standardization to zero mean and unit standard deviation.

### 4.2. Molecular Analysis of Hybridization

We obtained tissue samples (ventral scale clippings or shed skin) from 156 rattlesnakes, including field-caught specimens of *C. scutulatus*, *C. viridis* and *C. atrox* from throughout southern Arizona and New Mexico, and additional samples from colleagues and collaborators in the institutional and private sector. All field-collected specimens were released unharmed at the precise locality of capture within 72 h. Total genomic DNA was extracted using the DNeasy Blood and Tissue kit (Qiagen, Düsseldorf, Germany) following the manufacturer’s instructions.

For the assessment of hybridization, we PCR-amplified one mitochondrial gene fragment (NADH dehydrogenase subunit 4—hereafter ND4), and four single-copy nuclear loci: neurotrophin 3 (NT3, S. Cremer, unpublished), R35 [58], ETS oncogene [59], and SELT [60]. PCR was carried out in final volumes of 15 μL containing 1× ReddyMix PCR Master Mix (Thermo Scientific, Waltham, MA USA), 0.3 μM forward and reverse primers, and 1 μL of genomic DNA (approximate concentration 20 ng/μL). Primers and cycling conditions are given in Appendix A. Sanger chain termination direct sequencing was carried out at Macrogen, Seoul, South Korea. Nuclear loci were sequenced using both sense and antisense primers.

Sequence traces were checked for quality and aligned in CodonCode Aligner version 3.7.1 (CodonCode Corporation, Centerville, MA, USA). All coding sequences were translated to check that no frameshift mutations or unexpected stop codons were present. Heterozygous positions in nuclear sequences were identified by a combination of visual inspection for double peaks and typically low quality Phred scores [61] for the bases surrounding a heterozygous position. The individual alleles of length heterozygotes (in SELT only) were reconstructed using the online utility Indelligent v. 1.2 [62]. Individual allele sequences (haplotypes) were estimated from diploid nuclear loci using the software PHASE v. 2.1.1 [63,64] over 10,000 iterations with a burn-in of 5000 and a thinning interval of 100 for ETS, R35 and SELT, and 30,000, 15,000 and 300, respectively, for NT3, after preparation of the sequence data using SEQPHASE [65]. PHASE was run three times to confirm burn-in and convergence across multiple runs, and the highest probability haplotype pair for each specimen was retained for further analysis.

We determined the matrilineal line of descent of each specimen through a neighbor-joining tree of mitochondrial ND4 sequences. The analysis was run in MEGA 6.0.6 [66], using the maximum composite likelihood distance model, pairwise deletion of sites with missing base pairs, and 1000 bootstrap replicates. To visualize patterns of hybridization between species, we analyzed the nuclear genes with the Bayesian clustering method implemented in the software Structure 2.3.4 [67,68]. The analysis was run using an admixture model and both independent and correlated allele frequency models [69]. In the initial analyses run under inclusion of *C. atrox*, K was set to 3, reflecting the presence of three species in the dataset. After exclusion of *C. atrox*, the analysis was rerun solely on samples of *C. scutulatus* and *C. viridis* and likely hybrids between them, with K set to 2. All analyses were run over 100,000 iterations after a burn-in of 100,000 iterations, and in triplicate to check the consistency of results.

### 4.3. Determination of Mojave Toxin Presence

We used PCR amplification to test for the presence of the genes encoding the basic and acidic subunits of Mojave toxin in all specimens. Since the basic subunit primers from Wooldridge *et al.* [28] resulted in the amplification of PLA_2_s other than Mojave toxin in some MTX-negative individuals, we designed novel primers extending from exon 2 to exon 4 of the published MTX sequences of John *et al.* [53] (Genbank—acidic: U01026; basic: U01027). We verified the identity of all positive PCR products by Sanger sequencing (Macrogen, Seoul, South Korea). Primers and PCR conditions are given in Appendix A. Verification and alignment of the sequences against the published MTX sequences followed the procedures outlined for single copy nuclear genes above. Since even minimal contamination across samples can lead to false positive PCR results, anomalous or inconsistent results were checked by PCR amplification from fresh DNA extracts.

### 4.4. Determination of Mojave Toxin in the Venom

Approximately 0.7 mg of crude venoms from 41 specimens were separated by reverse-phase HPLC using a Teknokroma Europa 300 C18 column (250 × 4 mm, 5 µm particle size, Teknokroma, Barcelona, Spain) eluting at 1ml/min with a linear gradient of 0.1% TFA in water and acetonitrile. Presence of the two MTX subunits was determined by comparing our chromatograms to those published in Massey *et al.* [5].

## Figures and Tables

**Table 1:** Sequence characteristics of ND4 and single copy nuclear gene sequence alignments used in this study.

Sequence parameters	ND4	NT3	R35	SELT	ETS
**Length (bp)**	635	538	531	346	653
**# haplotypes**	57	39	18	20	26
**# variable positions**	139	31	15	17	29

**Table 2:** Morphological characters used for assessment of hybridization. Terminology for head scales follows [45].

1. Number of internasals contacting rostral scale
2. Minimum number of scales separating posteriormost canthals
3. Minimum number of scales separating supraoculars
4. Number of scales contacting the inner edge of the supraoculars
5. Number of dark (defined as noticeably darker than body markings) bands on tail
6. Number of light (defined as noticeably lighter than body ground colour) bands on tail
7. Maximum width in dorsal scale lengths along a single scale row (excluding the vertebral row) of the posteriormost black band not contacting the rattle fringe.
8. Maximum width in dorsal scale lengths along a single scale row (excluding the vertebral row) of the light band anterior to 7.
9. Basal rattle segment entirely light (0), black (1) or partly light, partly black (0.5).

## Abbreviations

The following abbreviations are used in this manuscript:

MTX	Mojave toxin
PLA_2_	Phospholipase A_2_
RP-HPLC	Reverse-phase high performance liquid chromatography
SVMP	Snake venom metalloproteinases

## Appendix A

Primer sequences (Table A1, references in main text) and PCR conditions (Table A2).

**Table A1:** Primer names and sequences for each locus.

Locus	Forward	Reverse
ND4	ND4: CACCTATGACTACCAAAAGCTCATGTAGAAGC	Leu: CATTACTTTTACTTGGATTTGCACCA
		H12763V: TTCTATCACTTGGATTTGCACCA
NT3	NTF3_SC_F: CGAGGTTTTGCACTGGGAAT	NTF3_SC_R: GCATTTCTGTGTGGCATCCA
R35	R35_F: GACTGTGGAYGAYCTGATCAGTGTGGTGCC	R35_R: GCCAAAATGAGSGAGAARCGCTTCTGAGC
SELT	SELT_F: GTTATYAGCCAGCGGTACCCAGACATCCG	SELT_R: GCCTATTAAYACTAGTTTGAAGACTGACAG
ETS	ETS_F: CCATCAACAGACACACAGG	ETS_R: GTCTGCTTTTTACTTTGCG
MTXa	MTXa2_F: TGCGGGGAGAAGTGGTATTT	MTXa4_R: GCAATTTTCGGGCGAGAACC
MTXb	MTXb2_F: ACCTGCTGCAATTCAACAAGA	MTXb4_R: CGAGAGTCCGGGTAAAACAT

**Table A2:** PCR conditions for each locus.

PCR Parameter	ND4	NT3/R35	SELT/ETS	MTXa/MTXb
1. Initial denaturation	94°-2 m	94°-2 m	94°-2 m	94°-2 m
2. Denaturation	94°-30 s	94°-30 s	94°-30 s	94°-30 s
3. Annealing	57°-30 s	55°-1 m	47°-1 m	59°-30 s
4. Extension	72°-1 m	72°-1 m	72°-1 m	72°-1.5 m
5. No. Cycles (2–4)	40	35	35	35
6. Final extension	72°-5 m	72°-5 m	72°-5 m	72°-5 m
